# Alpha-lipoic acid supplementation corrects pathological alterations in cellular models of pantothenate kinase-associated neurodegeneration with residual PANK2 expression levels

**DOI:** 10.1186/s13023-023-02687-5

**Published:** 2023-04-12

**Authors:** Marta Talaverón-Rey, Mónica Álvarez-Córdoba, Irene Villalón-García, Suleva Povea-Cabello, Juan M. Suárez-Rivero, David Gómez-Fernández, Ana Romero-González, Alejandra Suárez-Carrillo, Manuel Munuera-Cabeza, Paula Cilleros-Holgado, Diana Reche-López, Rocío Piñero-Pérez, José A. Sánchez-Alcázar

**Affiliations:** Centro Andaluz de Biología del Desarrollo (CABD-CSIC-UPO), Universidad Pablo de Olavide, 41013 Seville, Spain

**Keywords:** Pantothenate kinase, PANK2, Pantothenate kinase-associated neurodegeneration, PKAN, Coenzyme A, Mitochondria, α-lipoic acid, Induced neurons, Acyl carrier protein, 4′-phosphopantetheinylation

## Abstract

**Background:**

Neurodegeneration with brain iron accumulation (NBIA) disorders are a group of neurodegenerative diseases that have in common the accumulation of iron in the basal nuclei of the brain which are essential components of the extrapyramidal system. Frequent symptoms are progressive spasticity, dystonia, muscle rigidity, neuropsychiatric symptoms, and retinal degeneration or optic nerve atrophy. One of the most prevalent subtypes of NBIA is Pantothenate kinase-associated neurodegeneration (PKAN). It is caused by pathogenic variants in the gene of pantothenate kinase 2 (PANK2) which encodes the enzyme responsible for the first reaction on the coenzyme A (CoA) biosynthesis pathway. Thus, deficient PANK2 activity induces CoA deficiency as well as low expression levels of 4′-phosphopantetheinyl proteins which are essential for mitochondrial metabolism.

**Methods:**

This study is aimed at evaluating the role of alpha-lipoic acid (α-LA) in reversing the pathological alterations in fibroblasts and induced neurons derived from PKAN patients. Iron accumulation, lipid peroxidation, transcript and protein expression levels of PANK2, mitochondrial ACP (mtACP), 4′′-phosphopantetheinyl and lipoylated proteins, as well as pyruvate dehydrogenase (PDH) and Complex I activity were examined.

**Results:**

Treatment with α-LA was able to correct all pathological alterations in responsive mutant fibroblasts with residual PANK2 enzyme expression. However, α-LA had no effect on mutant fibroblasts with truncated/incomplete protein expression. The positive effect of α-LA in particular pathogenic variants was also confirmed in induced neurons derived from mutant fibroblasts**.**

**Conclusions:**

Our results suggest that α-LA treatment can increase the expression levels of PANK2 and reverse the mutant phenotype in PANK2 responsive pathogenic variants. The existence of residual enzyme expression in some affected individuals raises the possibility of treatment using high dose of α-LA.

**Supplementary Information:**

The online version contains supplementary material available at 10.1186/s13023-023-02687-5.

## Background

Neurodegeneration with Brain Iron Accumulation (NBIA) is a heterogeneous group of inherited diseases characterized by progressive neurodegeneration and abnormal iron deposition in the brain, mainly in globus pallidus and substantia nigra [1, 2]. NBIA disorders comprise 15 different subtypes [3], including pantothenate kinase-associated neurodegeneration, PLA2G6-associated neurodegeneration (PLAN) and β-propeller-associated neurodegeneration [4] as the most common [4].

Pantothenate kinase-associated neurodegeneration (PKAN) is an autosomal recessive disease caused by pathogenic variants in the pantothenate-kinase 2 gene (*PANK2*) [5]. *PANK2* encodes for a mitochondrial pantothenate kinase (PANK2) involved in the coenzyme A (CoA) biosynthesis pathway catalyzing the phosphorylation of pantothenate [6–9].

The main consequence of PANK2 alteration is a dysregulation in CoA homeostasis, which has secondary effects on cellular metabolism such as mitochondrial dysfunction, iron homeostasis disruption, lipid metabolism dysregulation or an impaired antioxidant system [10, 11].

CoA deficiency in PKAN affects posttranslational modifications of mitochondrial enzymes that need a 4′-phosphopantetheine cofactor [12]. As a consequence, expression levels of essential 4′-phosphopantetheinyl proteins in mitochondria—such as mitochondrial ACP (mtACP) in type II mitochondrial Fatty Acid Synthesis (mtFAS II), α-Aminoadipate semialdehyde synthase (AASS) in lysine metabolism and mitochondrial 10-formyltetrahydrofolate dehydrogenase (10-FTHFDH or ALDH1L2) involved in folate metabolism [13]- are markedly reduced.

PKAN is also characterized by an increase in oxidative stress and reactive oxygen species (ROS) production leading to lipid peroxidation/iron accumulation and eventually neuronal death by ferroptosis [14, 15]. Although neurons have mechanisms to counteract the effect of oxidative stress and ROS, it has been observed that they are downregulated in neurodegenerative diseases [16]. Among the cellular antioxidant systems, it has been described NRF2 as a transcription factor that regulates the synthesis of several antioxidant proteins such as catalase, superoxide dismutase (SOD) or glutathione peroxidases (GPX). NRF2 has a protective effect on neurodegenerative diseases and its activation through antioxidants treatment could be an alternative to curb the disease progression [17, 18]. In this respect, it has been described that antioxidant treatments may be beneficial for neurodegenerative disorders such as PLAN, Friedreich’s ataxia, or Alzheimer’s disease [19–23]. In addition, it has been shown that lipid peroxidation induction promotes iron accumulation in cellular models of NBIA [22]; in turn, ROS overproduction due to iron accumulation induces lipid peroxidation in a negative series of events that build on and reinforce each other [22]. Both processes promote lipofuscin accumulation which is associated with ageing and neurodegeneration [22, 24].

Unfortunately, existing treatments for PKAN are primarily palliative to pharmacologically treat the main symptoms of the disease. Recently, it has been reported that several commercial supplements (pantothenate, pantethine, vitamin E, omega 3, carnitine and thiamine) were able to eliminate iron accumulation, increase PANK2 and mtACP, and improve pathological alterations in mutant cells with residual PANK2 expression levels [25].

The main goal of this work was to evaluate the beneficial effect of alpha-lipoic acid (α-LA), another well-known nutritional supplement, on cellular models derived from PKAN patients. α-LA is an essential cofactor for mitochondrial metabolism with powerful antioxidant properties and promising therapeutic benefits in preventing or treating various diseases, including neurodegenerative diseases [26]. Thus, α-LA has been reported to reduce mitochondrial dysfunction, ROS formation and neuronal damage [27]. The potential therapeutic utility of α-LA in PKAN is also discussed.

## Methods

### Reagents

Sudan Black, Prussian Blue, ( ±) α-LA, Luperox® DI (tert-Butyl peroxide), anti-fatty acid synthase (FAS), and trypsin were purchased from Sigma Chemical Co. (St. Louis, MO). BODIPY® 598/591 C11, MitoTracker Deep Red FM, DAPI, were purchased from Invitrogen/Molecular Probes (Eugene, OR). MitoPeDPP® was purchased from Dojindo Molecular Technologies, Inc. (Rockville,MD) Anti-PANK2, anti-MTND1, anti-NDUFA9, anti-NFS1, anti-ISCU, anti-LYRM4anti-NRF2, PDH hand complex I activity kit and aconitase kit were purchased from Abcam (Cambridge, UK), Anti-mitochondrial 10-formyltetrahydrofolate dehydrogenase (ALDH1L2), anti-alpha-aminoadipic semialdehyde synthase (AASS), anti-FOXN4, anti-hnRNPA/B,anti-NF-Y, anti-Tau, anti-GPX4 and anti-AASDHPPT were purchased from Thermo-Fisher (Waltham, MA). Anti-lipoic acid was acquired from Merck (Darmstadt, Germany). Anti-PLA2G6 and anti-SOD were purchased from Santa Cruz Biotechnology (Dallas, TX, USA). Anti-actin was acquired from MyBiosource (San Diego, California, USA). OxyBlot Protein Oxidation Detection Kit was acquired from Merck (Darmstadt, Germany). A cocktail of protease inhibitors (complete cocktail) was purchased from Boehringer Mannheim (Indianapolis, IN). The Immun Star HRP substrate kit was from Bio-Rad Laboratories Inc. (Hercules, CA).

### Ethical statements

Approval of the ethical committee of the Hospital Universitario Virgen Macarena y Virgen de Rocío de Sevilla (Spain) was obtained, according to the principles of the Declaration of Helsinki and all the International Conferences on Harmonization and Good Clinical Practice Guidelines.

### Cell culture

Three lines of fibroblasts derived from patient skin biopsies from the Movement Disorder Unit of Hospital Universitario Virgen del Rocío, Sevilla, Spain, and from the Movement Disorders Bio-Bank available at the Neurogenetics Unit of the Neurological Institute ‘Carlo Besta’ (INCB), Milan, Italy and three controls lines of primary human skin fibroblasts were purchased from ATCC. Patient 1 (P1 presents a heterozygous pathogenic variant c.747dup (p.Arg249ProfsX43) that causes a stop codon, and a heterozygous variant c.1475C > G (p.Ala492Gly) that causes a missense variant which is predicted to be damaging by prediction tools such as PolyPhen2 [28]. The second patient (P2) is also heterozygous carrying changes in position 240-241delCA (p.Tyr80_Ser81delinsTer) and 650C > T (ThrT217Ile) which have been previously described [29]. The third patient (P3) P3 presents a homozygous pathogenic variant c.1259delG causing a frameshift p.Gly420Valfs*30 [14]. Control values were represented as means ± SD of three control lines. Fibroblasts were grown in Dulbecco's modified Eagle's medium DMEM (Gibco™, ThermoFisher Scientific, Waltham, MA, USA) supplemented with 10% FBS (Gibco™, ThermoFisher Scientific, Waltham, MA, USA), 100 mg/ml penicillin/streptomycin. Fibroblasts were cultured at 37ºC and 5% CO_2._ iNs were cultured in Neuronal Differentiation medium (NDiff227; Takara-Clontech, Kusatsu, Prefecture of Shiga, Japan) supplemented with neural growth factors and small molecules at different concentration [30]_._ Experiments were performed with less than 12 passage fibroblasts cultures. Patients and controls fibroblasts were treated with 10 μM of α-LA for twenty days.

### Iron and lipofuscin accumulation

Iron accumulation was assessed by Perls' Prussian blue staining [31]. Images were taken by light and fluorescence microscopy Axio Vert A1 microscope (Zeiss, Oberkochen, Germany) and analyzed by FIJI-ImageJ software. Iron content was also examined by colorimetric Ferrozine-based assay [32]

Lipofuscin accumulation was determined by Sudan Black B (SBB) staining as previously described [33]. SSB staining quantification was assessed by light microscopy.

### Immunoblotting

Western blotting was performed using standard Methods. After transferring protein to a nitrocellulose membrane. The membrane was incubated with primary antibodies diluted 1:1000, and then with the corresponding secondary antibody coupled to horseradish peroxidase at a 1:2500 dilution. Specific protein complexes were identified by ChemiDoc™ MP Imaging System (Bio-Rad, Hercules, CA, USA) using the Immun Star HRP substrate kit (Biorad Laboratories Inc., Hercules, CA, USA). ImageLab™ version 5.0 software (Bio-Rad, Hercules, CA, USA) was used to analyze protein expression levels.

### Immunofluorescence microscopy

iNs were plated on μ-Slide 4 well (Ibidi Inc., Martinsried, Germany). Cells were rinsed once with PBS, fixed in 3.8% paraformaldehyde for 10 min at room temperature. Then, cells were permeabilized with Triton X-100 0,1% for 10 min. Next, cells were incubated with blocking solution 5% donkey serum for 1 h. Primary antibodies diluted 1:200–1:500 in blocking solution were incubated overnight at 4 °C. Unbound antibodies were removed by washing twice with PBS. Cells were incubated with secondary antibodies, diluted 1:300 in blocking solution, for 2 h at room temperature. Finally, cells were stained with 1 μg/mL DAPI for 15–20 min. Images were taken with a DeltaVision system with an Olympus IX-71 fluorescence microscope with a 60 × oil objective and analysed by Fiji-ImageJ software.

### Oxidative stress analysis

Oxidized proteins were detected using the Oxyblot Protein Oxidation Detection Kit following the manufacturer’s instructions. Lipid peroxidation was evaluated using 4,4-difluoro-5-(4-phenyl-1,3-butadienyl)-4-bora-3a,4a-diaza-s-indacene-3-undecanoic acid (BODIPY® 581/591 C11), a lipophilic fluorescent dye [34, 35]. Cells were incubated with 1–5 µM BODIPY® 581/591 C11 for 30 min at 37 °C. Control fibroblasts treated with 500 µM Luperox® for 15 min were used as positive control of lipid peroxidation. Lipid peroxidation in fibroblasts was evaluated by an Axio Vert A1 fluorescence microscope with a 20X objective. Images were analysed with Fiji-ImageJ software.

Mitochondrial lipid peroxidation was determined using [3-(4-phenoxyphenylpyrenylphosphino) propyl]triphenylphosphonium iodide fluorescent probe (MitoPeDPP®) developed by Shioji et al. [36] Fibroblasts were treated with 300 nM MitoPeDPP® and 100 nM MitoTracker™ Deep Red FM, Cells were incubated with Luperox® for 15 min to induce a positive control. Images were taken in vivo at DeltaVision system with an Olympus IX-71 fluorescence microscope with 20 × objective and analysed by Fiji-ImageJ software.

### Real-time quantitative PCR (qPCR)

*PANK2* gene expression in fibroblasts was analysed by qPCR using mRNA extracts. mRNA was isolated with Trizol™ (Invitrogen, Carlsbad, CA, USA), following manufacturer’s instructions. RNA was retrotranscribed using Iscript cDNA synthesis Kit (Bio-Rad,Hercules, CA, United States) to obtain complementary DNA (cDNA). qPCR was performed using TB Green™ Premix Ex Taq™ (Takara Bio Europe S.A.S., Saint-Germain-en-Laye, France). CFX Connect Real-Time PCR Detection System (Bio-Rad, Hercules, CA, USA) was used to detect accurate quantification of gene expression. PANK2 primers were 5′ TTCCCACTCATGACATGCCT-3′ (Forward primer) and 5′-GTGACCGTCCATTGAATCCG-3′ (Reverse primer) amplifying a sequence of 215 nucleotides. Actin was used as a housekeeping control gene and the primers were 5′-AGAGCTACGAGCTGCCTGAC-3′ (Forward primer) and 3′-AGCACTGTGTTGGCGTACAG-5′ (reverse primer).

### Complex I activity

Complex I activity in whole cells was measured using the Complex I Enzyme Activity Dipstick Assay Kit (ab109720, ABCAM, Cambridge, MA, USA) according to manufacturer’s instructions. Three biological replicates were used per measurement. Results are expressed as enzyme activity with respect to control. The signal intensity was analyzed by a Molecular Imager ChemiDoc™ MP Imaging System (Bio-Rad, Hercules, CA, USA).

### PDH activity

PDH complex activity in whole cells was measured using the Pyruvate dehydrogenase (PDH) Enzyme Activity Dipstick Assay Kit (ab109882, ABCAM, Cambridge, MA, USA) according to manufacturer’s instructions. Three biological replicates were used per measurement. Results are expressed as enzyme activity with respect to control. The signal intensity was analyzed by a Molecular Imager ChemiDoc™ MP Imaging System (Bio-Rad, Hercules, CA, USA).

### Generation of induced neurons from PKAN (P1) fibroblasts by direct reprogramming

Neurons were generated from PKAN and control fibroblasts by direct reprogramming as previously described by Drouin-Ouellet et al. [37–39]. Controls and patients-derived fibroblasts were seeded in µ-Slide 4 Well (Ibidi Inc., Martinsried, Germany). The day after, dermal fibroblasts were infected with one-single lentiviral vector containing neural transcription factors (Acsl1 and Brn2) and two shRNA against the REST complex, generated as previously described [40]. Cells were infected with a multiplicity of infection (MOI) of 30. The plasmids were a gift from Dr. Malin Parmar (Developmental and Regenerative Neurobiology, Lund University, Sweden). After 24 h, medium was replaced with fresh fibroblast medium. Fibroblasts medium was replaced with neural differentiation medium after 48 h (NDiff227; Takara-Clontech) as described. Twenty-seven days post-infection neuronal cells were identified by the expression of Tau. DAPI + and Tau + cells were considered induced neurons. Images were taken at DeltaVision system with an Olympus IX-71 fluorescence microscope with 60 × oil objective and analysed by Fiji-ImageJ software.

### Statistical analyses

Statical analysis was routinely performed as formerly described by our research group [41]. We used non-parametric statistics that do not have any distributional assumption in cases when the number of events was small (n < 30) [42]. In these cases, multiple groups were compared using a Kruskal–Wallis test. In cases when number of events was higher (n > 30), we applied parametric tests. In these cases, multiple groups were compared using a one-way ANOVA. Statistical analyses were conducted using the GraphPad Prism 9.0 (GraphPad Software, San Diego, CA). The data are reported as the mean ± SD values or as representative of at least three independent experiments. *P*-values of less than 0.05 were considered significant.

## Results

### α-LA supplementation reduces iron accumulation and increases PANK2 and mtACP expression levels in a dose-dependent manner in mutant fibroblasts with residual enzyme activity

In previous work, we analyzed the PANK2 enzyme expression levels in skin fibroblasts derived from three different PKAN patients [12, 43]. Two PKAN patients, patient 1 (P1) and patient 2 (P2), carried double heterozygous pathogenic variants showing low residual PANK2 expression levels, while patient 3 (P3) carried a frameshift pathogenic variant in both alleles that results in a truncated PANK2 protein and ultimately, in a total absence of protein expression. Intracellular iron accumulation was observed in the three mutant cell lines [12, 43].

**Fig. 1:**
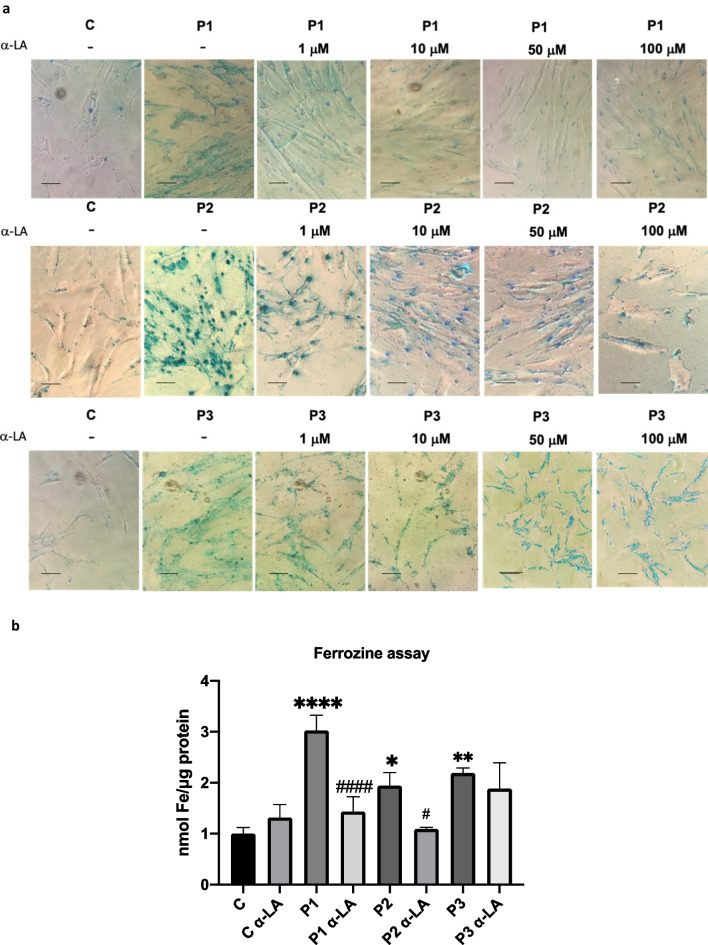
Effect of α-LA supplementation on iron accumulation in three mutant PANK2 cells. **a** Control (C1) and three PKAN fibroblast cell lines (P1, P2 and P3) were treated with increasing α-LA concentrations (1, 10, 50, 100 μM) for 20 days. Then, cells were stained with Prussian Blue as described in Material and Methods and examined by bright-field microscopy. Scale bar = 15 μm. Quantification of Prussian Blue staining is shown in Additional file 1: Fig. 1 **b** Iron accumulation determined by colorimetric Ferrozine-based assay. Significance between PKAN and control fibroblasts is represented as ****p < 0.0001, **p < 0.005, *p < 0.05 fibroblasts and ^####^*p* < 0.0001, ^#^*p* < 0.05 between untreated and treated fibroblasts

First, to assess the effect of α-LA on iron accumulation, Control and PKAN fibroblasts (P1, P2 and P3) were treated with increasing doses of α-LA (1 μM, 10 μM, 50 μM, 100 μM) and Prussian Blue staining was performed. As it is illustrated in Fig. 1a and Additional file 1: Fig. S1, iron accumulation was markedly reduced with α-LA supplementation in a dose-dependent manner in P1 and P2 but not in P3 fibroblasts. The positive effect of α-LA (10 μM) on intracellular iron accumulation in PKAN fibroblasts was confirmed by a colorimetric Ferrozine-based assay (Fig. 1b).

**Fig. 2:**
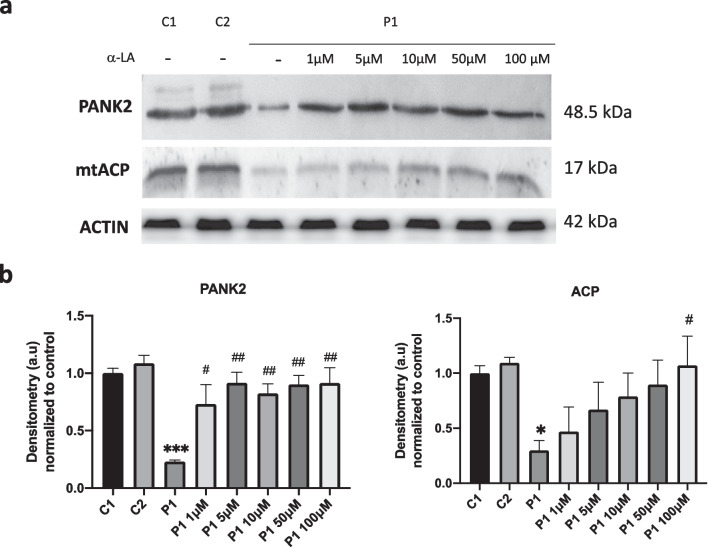
Dose–response effect of α-LA on PANK2 and mtACP protein expression levels. **a** Controls (C1, C2) and patient P1 fibroblasts were treated with increasing concentrations of lipoic acid for 20 days. PANK2 and mtACP protein expression levels of P1 levels were analysed by Western blotting. Actin was used as loading control. **b** Densitometry of the Western blotting of PANK2 and mtACP. Data represent the mean ± SD of three separate experiments. *p < 0.05, ***p < 0.001 between PKAN patients and controls. ^#^*p* < 0.05 and ^##^*p* < 0.005 between untreated and treated fibroblasts. A.U., arbitrary units

To address if the positive effect of α-LA on iron accumulation in responsive fibroblasts cell lines was associated with PANK2 and mtACP up-regulation, P1 fibroblasts were treated with increasing doses of α-LA and the expression levels of these proteins were examined by Western blotting. As shown in Fig. 2a, α-LA supplementation increased the expression levels of both PANK2 and mtACP proteins in P1 fibroblasts in a dose-dependent manner. The positive effect of α-LA supplementation on both PANK2 and mtACP expression levels was observed since 10 μM**.**

### α-LA activates ***PANK2*** gene expression

**Fig. 3:**
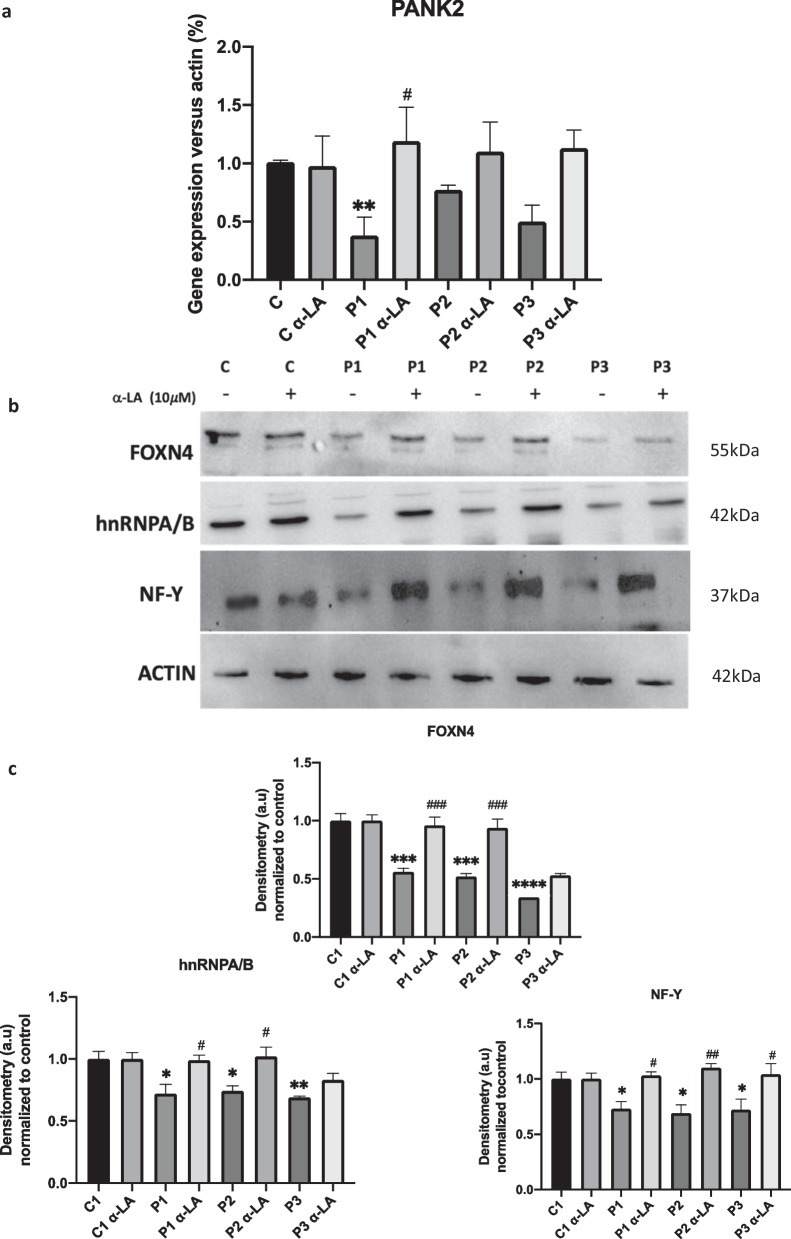
Effect of α-LA supplementation on PANK2 gene expression. Control and PKAN fibroblasts (P1, P2, P3) were treated with lipoic acid at 10 μM for twenty days. **a** PANK2 transcripts were quantified by RT-qPCR. **b** NF-Y, FOXN4 and hnRNPA/B transcription factors expression levels were analyzed by Western blotting. Actin was used as a loading control. **c** Densitometry of Western blotting. *p < 0.05, **p < 0.01, ***p < 0.005 ****p < 0.001 between PKAN patients and controls. ^#^*p* < 0.05, ^##^*p* < 0.01, ^###^*p* < 0.005 between untreated and treated fibroblasts. A.U., arbitrary units

Next, to assess if α-LA supplementation had a positive effect at the transcriptional level, we examined PANK2 RNA expression levels by RT-qPCR. PANK2 RNA levels were significantly reduced in P1, P2 and P3 mutant fibroblasts (Fig. 3a). Interestingly, α-LA treatment increased PANK2 RNA levels in the three PKAN fibroblasts cell lines. To support these results, we next examined the expression levels of three transcription factors that have been associated with *PANK2* expression [44]. Under α-LA supplementation, expression levels of FOXN4, hnRNPA/B and NF-Y were notably increased in all three PKAN fibroblast cell lines (Fig. 3b).

### α-LA supplementation increases mitochondrial 4′-phosphopantetheinyl proteins expression levels in mutant fibroblasts with residual enzyme levels

**Fig. 4:**
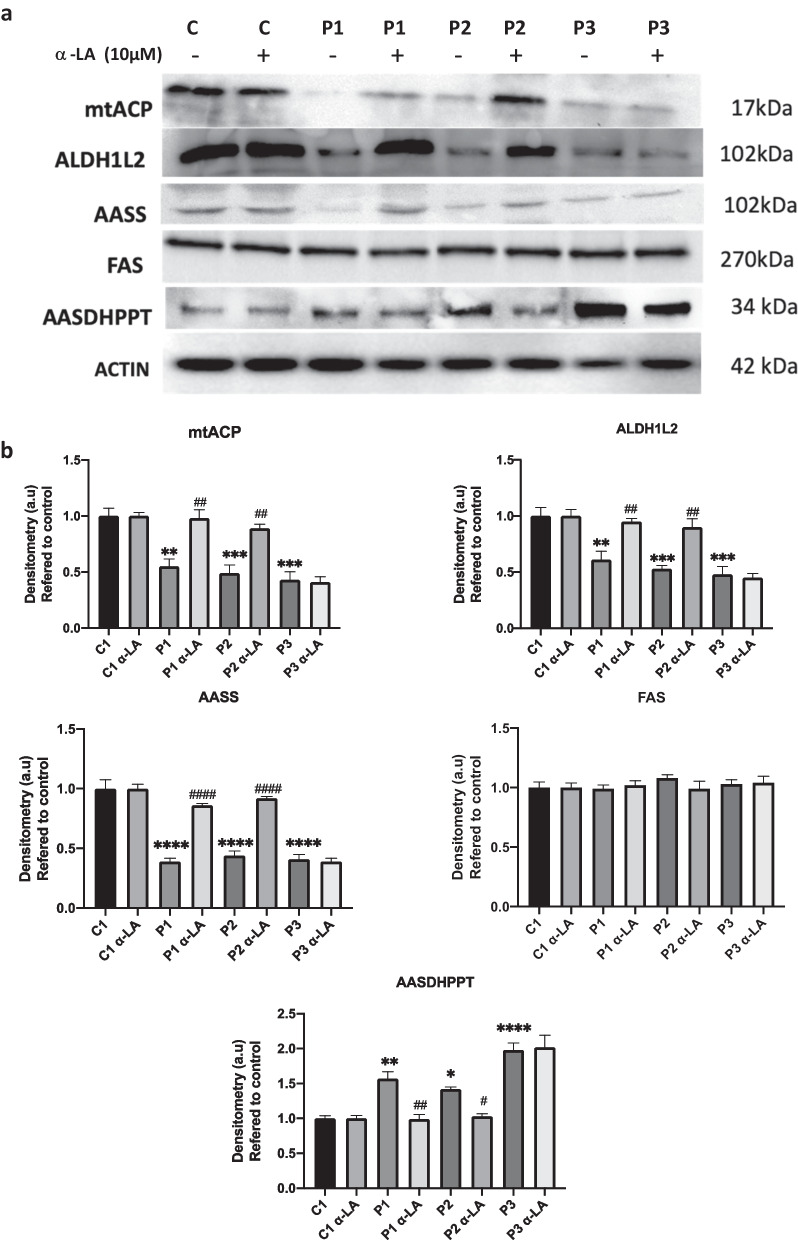
Effect of α-LA treatment on 4′-phosphopantetheinyl proteins expression levels. **a** Control and PKAN fibroblasts (P1, P2, P3) were treated with α-LA at 10 μM for twenty days. Protein extracts were separated on a SDS polyacrylamide gel and immunostained with antibodies against mtACP, ALDH1L2, AASS, FAS and AASDHPPT. Actin was used as a loading control. **(b)** Densitometry of Western blotting. *p < 0.05, **p < 0.005, ***p < 0.001 ****p < 0.0001 between PKAN patients and controls. ^#^*p* < 0.05, ^##^*p* < 0.005, ^###^*p* < 0.001 ^####^*p* < 0.0001 between untreated and treated fibroblasts. A.U., arbitrary units

Recently, our group described that mitochondrial phosphopantetheinyl-proteins are downregulated in PKAN patient-derived fibroblasts [12]. To evaluate if α-LA has an effect on this alteration, we treated the three PKAN fibroblast cell lines with α-LA and performed a Western blot assay. As it is shown in Fig. 4, expression levels of mitochondrial 4′-phosphopantetheine carrier proteins such as mtACP, ALDH1L2 and AASS were markedly reduced in PKAN patient-derived fibroblasts in comparison to controls. Interestingly, α-LA treatment significantly restored the expression levels of 4′-phosphopantetheinyl proteins in PKAN fibroblasts, P1 and P2, with residual expression levels, but not in P3 fibroblasts harbouring a homozygous nonsense pathogenic variant that results in a truncated protein product. In contrast, the expression levels of AASDHPPT, the enzyme in charge of transferring phosphopantetheine from CoA to target proteins, were markedly increased in all PKAN fibroblast cell lines, presumably to compensate for the low levels of CoA. As expected, α-LA treatment corrected the expression levels of AASDHPPT in responsive pathogenic variants (P1 and P2) but not in non-responsive fibroblasts (P3) (Fig. 4a).

### Mitochondrial complex I function improves after α-LA treatment in responsive PKAN fibroblasts

**Fig. 5:**
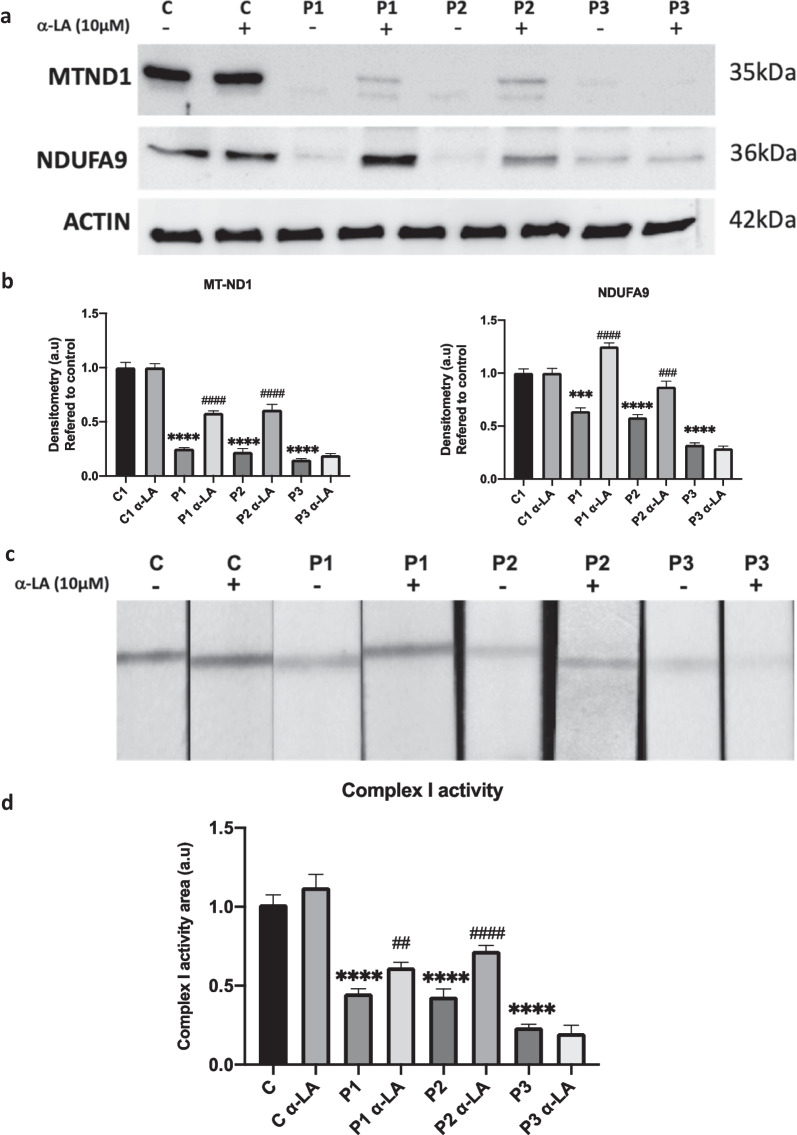
Effect of α-LA on mitochondrial complex I. Control and PKAN fibroblasts (P1, P2, P3) were treated with α-LA at 10 μM for twenty days. **a** MT-ND1 and NDUFA9, mitochondrial complex I subunits, analysed by Western blotting. **b** Densitometry of Western blotting. **c** Mitochondrial complex I activity in whole cellular extracts was measured as described in Material and Methods. **d** Quantification of mitochondrial complex I activity. Data represent the mean ± SD of two separate experiments. **p < 0.005, ***p < 0.001 ****p < 0.0001 between PKAN patients and controls; ^##^*p* < 0.005, ^###^*p* < 0.001 ^####^*p* < 0.0001 between untreated and treated cells

Given that mtACP is also directly involved in mitochondrial complex I assembly [45], we next analyzed if mtACP expression correction by α-LA was also able to improve complex I activity that is reduced in PKAN fibroblasts [12]. We also quantified the expression levels of MT-ND1 and NDUFA9, two proteins forming part of Complex I. As it is shown in Fig. 5a, α-LA supplementation induced an increase in the expression of MT-ND1 and NDUFA9 that were markedly reduced in PKAN fibroblasts. In addition, complex I enzymatic activity was partially rescued by α-LA in responsive mutant cells P1 and P2 but not in P3 (Fig. 5c).

### Iron-sulfur cluster biosynthesis disruption is corrected by α-LA supplementation

**Fig. 6:**
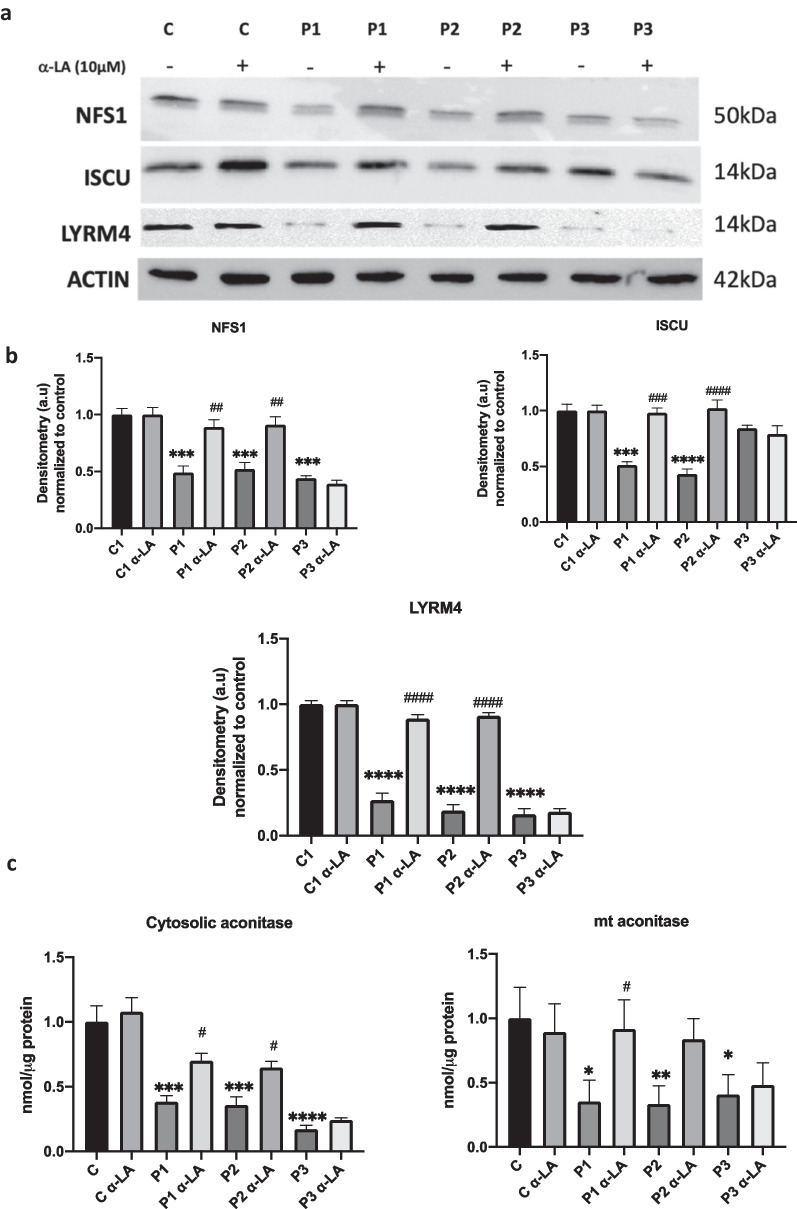
Effect of α-LA treatment on Fe-S cluster assembly complex proteins and aconitase activity. Control and PKAN fibroblasts (P1, P2, P3) were treated with α-LA at 10 μM for 20 days. a Representative image of NFS1, ISCU and LYRM4 protein levels, proteins involved in Fe-S cluster assembly, analysed by Western blotting of treated and untreated control and PKAN fibroblasts. **b** Densitometry of Western blotting. **c** Both mitochondrial and cytosolic aconitase activity were determined by colorimetric assay**.** Data represent the mean ± SD of three separate experiments. *p < 0.05, **p < 0.005, ***p < 0.005 ****p < 0.0001 between PKAN patients and controls; ^#^*p* < 0.05, ^##^*p* < 0.005, ^###^*p* < 0.001 between untreated and treated cells

The polyvalent protein, mtACP, is also involved in Fe/S cluster biogenesis assembly and stabilization [46, 47]. Consequently, other iron-sulfur complex proteins can be affected in PKAN fibroblasts. As expected, cysteine desulfurase (NFS1), iron-sulfur cluster assembly enzyme (ISCU) and LYR motif-containing protein 4 (LYRM4) expression levels were reduced (Fig. 6a, b). Interestingly, α-LA supplementation corrected NFS1, ISCU and LYRM4 protein expression levels in P1 and P2 fibroblasts but not in P3 fibroblasts. Additionally, we examined the effect of α-LA on both mitochondrial and cytosolic aconitase activity, an Fe-S dependent-enzyme [48]. Both cytosolic and mitochondrial aconitase activities were significantly reduced in PKAN mutant fibroblasts. As predicted, α-LA treatment was able to restore their activities to control levels only in responsive mutant cells P1 and P2 with residual PANK2 enzyme expression (Fig. 6c).

### α-LA supplementation increases the lipoylation of mitochondrial proteins

**Fig. 7:**
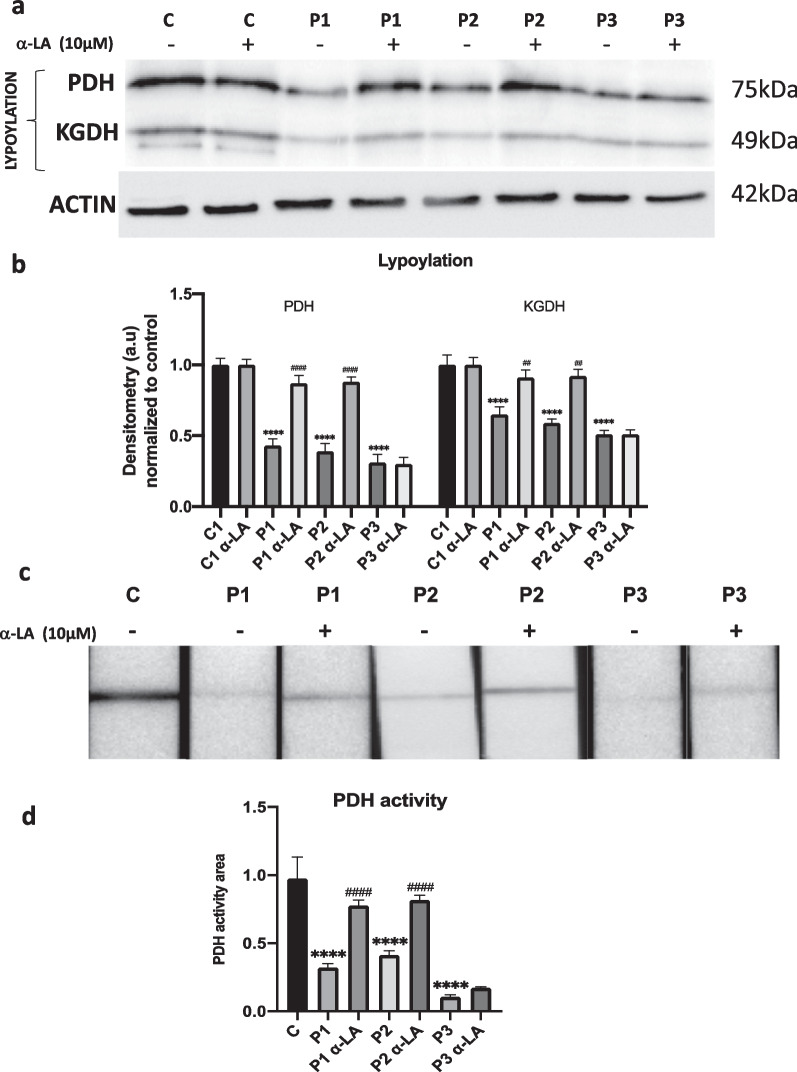
Effect of α-LA supplementation on mitochondrial lipoylated proteins and PDH activity. Control and PKAN fibroblasts (P1, P2, P3) were treated with α-LA at 10 μM for 20 days. **a** Representative image of lipoylated proteins expression levels assessed by Western blotting. **b** Densitometry of the Western blotting. **c** PDH activity in whole cellular extracts was measured as described in Material and Methods. Data represent the mean ± SD of two separate experiments. ****p < 0.0001 between PKAN patients and controls; ^##^*p* < 0.005, ^###^*p* < 0.001 ^####^*p* < 0.0001 between untreated and treated cells

Several studies support that α-LA biosynthesis by mtFAS II is essential for mitochondrial protein lipoylation [45, 49, 50]. Given that mtACP also participates in mtFAS II, the endogenous α-LA biosynthesis and protein lipoylation are downregulated processes in PKAN fibroblasts [12, 43]. Corroborating these results, lipoylation of pyruvate dehydrogenase (PDH) and alpha-ketoglutarate dehydrogenase (KGDH) were markedly reduced in PKAN fibroblasts (Fig. 7a). Interestingly, α-LA supplementation significantly increased PDH and KGDH lipoylation in P1 and P2 fibroblasts cell lines but not in P3 fibroblasts cell line (Fig. 7a, b). Consistently, α-LA supplementation also was also able to restore PDH activity in P1 and P2 fibroblasts but not in P3 fibroblasts (Fig. 7c, d).

### α-LA treatment recovers cells from oxidative stress

Iron accumulation and cellular metabolism dysregulation alter cellular oxidative status in PKAN cells [51]. It has been described that PKAN fibroblasts and iPSC derived neurons show lipid peroxidation, carbonylated proteins, deficient antioxidant system and altered mitochondria membrane potential [6, 52, 53]. To address the effect of α-LA on cellular oxidative stress in PKAN fibroblasts we perform several assays. First, cellular, and mitochondrial lipid peroxidation was evaluated by Bodipy and MitoPeDPP assays, respectively.

**Fig. 8:**
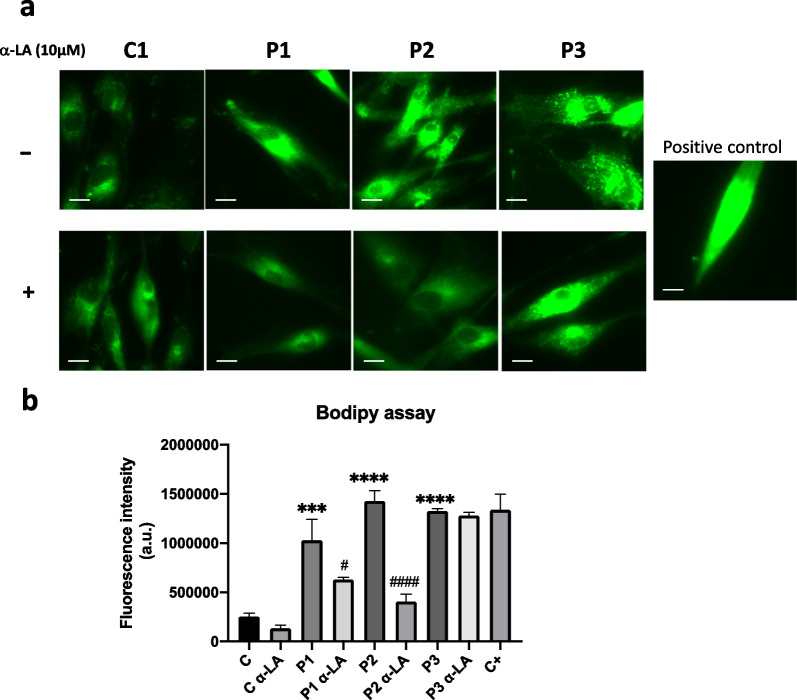
Effect of α-LA treatment on lipid peroxidation. Control and PKAN fibroblasts (P1, P2 and P3) were treated with α-LA at 10 μM for 20 days. **a** Representative images of lipid peroxidation in treated and untreated control and PKAN cells using BODIPY® 581/591 C11 staining. Control cells treated with Luperox® (500 μM) for 15 min were used as a positive control of mitochondrial lipid peroxidation. Scale bar = 15 μm. **b** Fluorescence quantification of oxidized form of BODIPY® C11. Data represent the mean ± SD of three separate experiments (50 cell images for each condition). ***p < 0.001, ****p < 0.0001 between PKAN patients and controls. ^#^*p* < 0.05, ^###^*p* < 0.001, between untreated and treated fibroblasts. A.U., arbitrary units

**Fig. 9:**
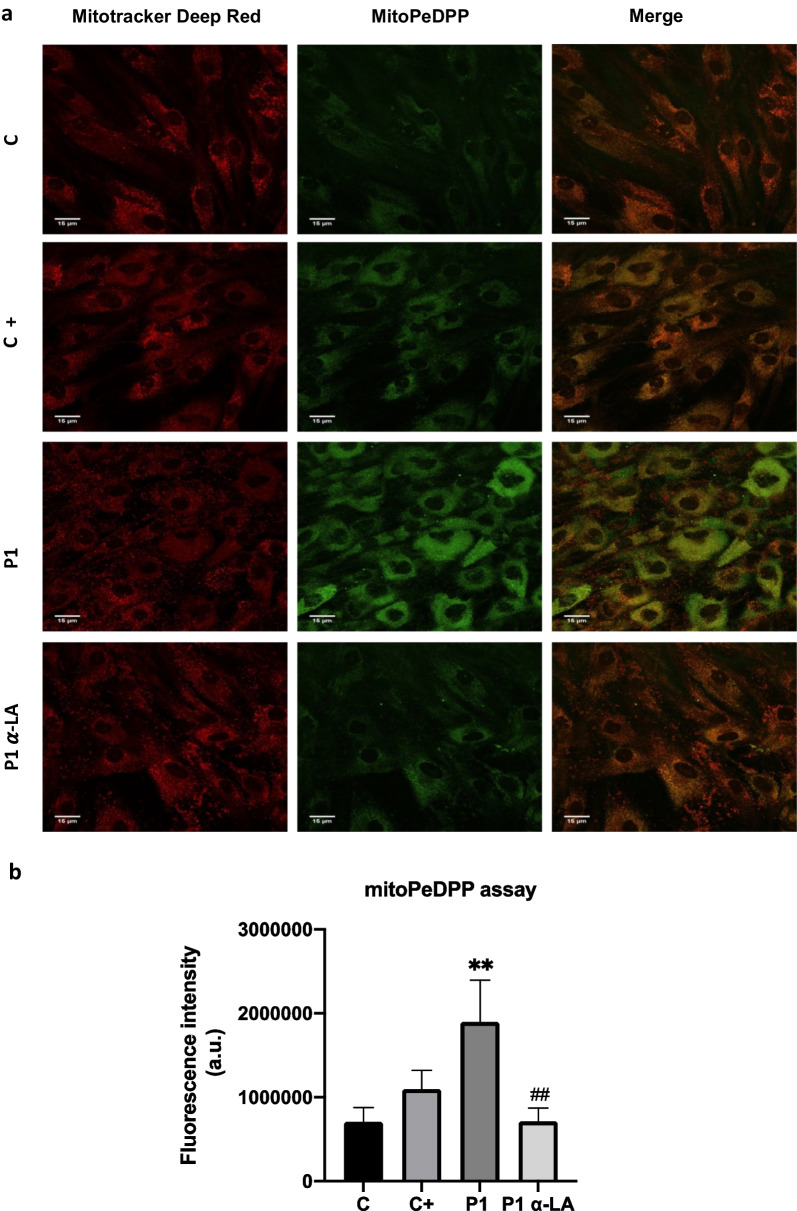
Effect of α-LA treatment on mitochondrial lipid peroxidation. Control and P1 PKAN fibroblasts were treated with α-LA at 10 μM for 20 days. **a** Representative images of mitochondrial lipid peroxidation in lipoic acid treated and untreated control and PKAN cells by MitoPeDPP staining. Scale bar = 15 μm. Cells also were stained with Mitotracker Deep Red. **b** Fluorescence quantification of MitoPeDPP. Control cells treated with Luperox® (500 μM) for 15 min were used as a positive control of mitochondrial lipid peroxidation. Data represent the mean ± SD of three separate experiments (50 cell images for each condition). *p < 0.01 between PKAN patients and controls. ^#^*p* < 0.01 between untreated and treated fibroblasts. A.U., arbitrary units

Both cellular and mitochondrial lipid peroxidation were notably increased in mutant PKAN cells. As expected, α-LA supplementation significantly reduced lipid peroxidation in cellular membranes in P1 and P2 fibroblasts (Fig. 8) and mitochondrial membranes (Fig. 9) in P1 with residual PANK2 expression levels. However, α-LA is not able to reduce cellular lipid peroxidation in P3 carrying a PANK2 truncated protein.

**Fig. 10:**
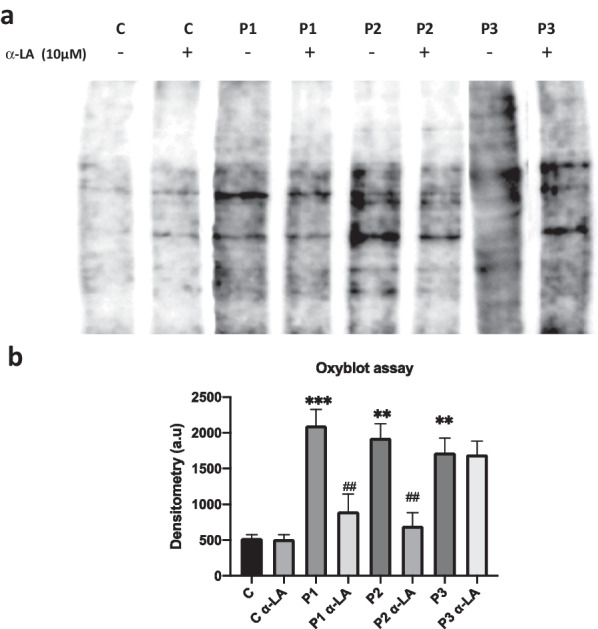
Effect of α-LA treatment on carbonylated protein levels. Control and PKAN fibroblasts (P1, P2, P3) were treated with α-LA at 10 μM for 20 days. **a** A representative image of carbonylated protein content in treated and untreated control and PKAN fibroblasts by Oxyblot Protein Oxidation Kit **b** Oxyblot quantification by ImageJ. Data represent the mean ± SD of three separate experiments. **p < 0.005 ***p < 0.001 between PKAN patients and controls. ^##^*p* < 0.005 between untreated and treated fibroblasts. A.U., arbitrary units

Furthermore, we measured protein oxidation in lipoic-treated and untreated cellular extracts using an Oxyblot assay. Carbonylated protein levels were notably increased in PKAN fibroblasts (Fig. 10). As predicted, α-LA supplementation significantly reduced protein oxidation levels in P1 and P2 but not in P3 fibroblasts (Fig. 10).

**Fig. 11:**
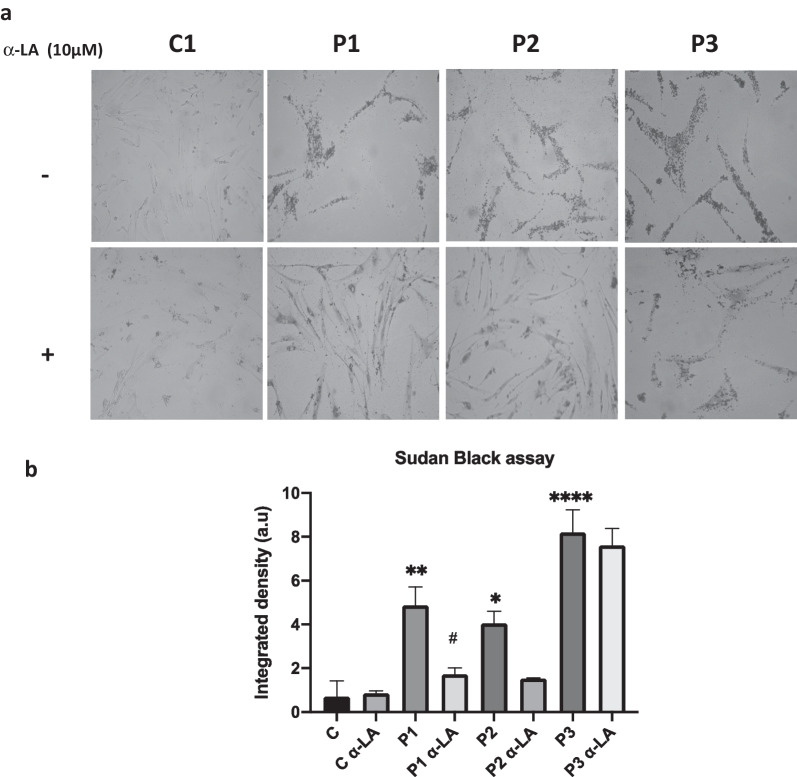
Effect of α-LA treatment on lipofuscin accumulation. Control and PKAN fibroblasts (P1, P2, P3) were treated with α-LA at 10 μM for 20 days. **a** Representative images of lipofuscin staining by SBB of untreated and treated control and three PKAN patient fibroblasts**.** Scale bar = 20 μm. **b** SBB staining quantification. Data represent the mean ± SD of three separate experiments (50 cell images for each condition). *p < 0.05, **p < 0.05, ****p < 0.001 between PKAN patients and controls. ^#^*p* < 0.05 between untreated and treated fibroblasts. A.U., arbitrary units

Additionally, lipofuscin accumulation was determined by Sudan Black B assay in treated and untreated PKAN fibroblasts. Results showed that α-LA was able to reduce lipofuscin accumulation (Fig. 11).

### α-LA activates cellular antioxidant system

**Fig. 12:**
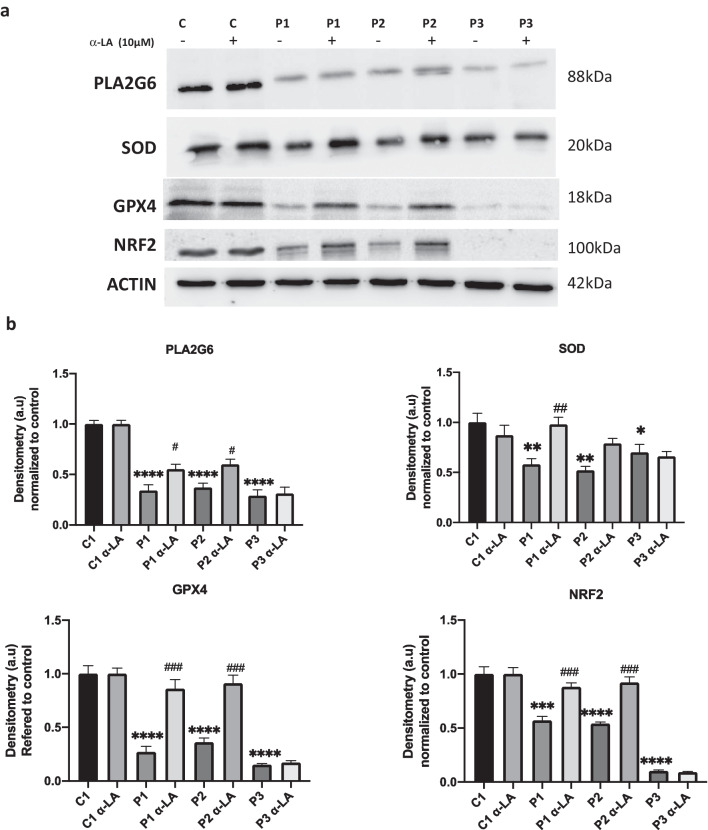
Effect of α-LA on antioxidant protein expression levels. Control and PKAN fibroblasts (P1, P2, P3) were treated with α-LA at 10 μM for 20 days **a** Expression levels of PLA2G6, SOD, GPX4 and NRF2 in treated and untreated control and PKAN cells. Actin was used as loading control. **b** Densitometry of the Western blotting. Data represent the mean ± SD of three separate experiments. **p < 0.01, ***p < 0.005, ****p < 0.0001 between PKAN patients and controls. ^#^*p* < 0.05, ^##^*p* < 0.01, ^###^*p* < 0.005 between untreated and treated fibroblasts. A.U., arbitrary units

Given that the antioxidant system is decreased in neurodegenerative diseases [7, 54, 55], we also examined the effect of α-LA on several antioxidant proteins such as GPX4, SOD or PLA2G6 in PKAN fibroblasts. As expected, α-LA restored GPX4 and SOD expression in responsive PKAN fibroblasts, but we did not observe any effect in the case of P3 suggesting that it was dependent on PANK2 expression. However, α-LA did not show any effect on PLA2G6 expression levels. We also assessed the expression levels of NRF2, a transcription factor involved in antioxidant proteins regulation [56]. NFR2 expression levels were downregulated in PKAN fibroblasts, and they were restored by α-LA supplementation in responsive mutant cells P1 and P2 with residual PANK2 expression but not in P3 with a truncated PANK2 protein expression (Fig. 12).

### α-LA supplementation also has beneficial effect on PKAN induced neurons (iNs)

To further demonstrate the beneficial effect of α-LA in PANK2 pathogenic variants with residual expression levels, control and patient responsive fibroblasts P1 were transdifferentiated to induced neurons by direct reprogramming. Thus, control and PANK2 mutant fibroblasts were infected with lentiviral vectors expressing proneural genes Ascl1 and Brn2 and also promoting the knock down of the REST complex [37]. After transdifferentiation, cells manifested a typical neuron-like morphology and showed positive immunoreactivity against Tau, a neuron-specific protein. In contrast, undifferentiated fibroblasts did not show Tau staining. Positive cells for Tau were used to evaluate neuronal conversion efficiency, which was approximately 55% in control cells and 25% in PANK2 mutant cells. Neuronal purity was almost 40% in control cells and 50% in PANK2 mutant cells.

**Fig. 13:**
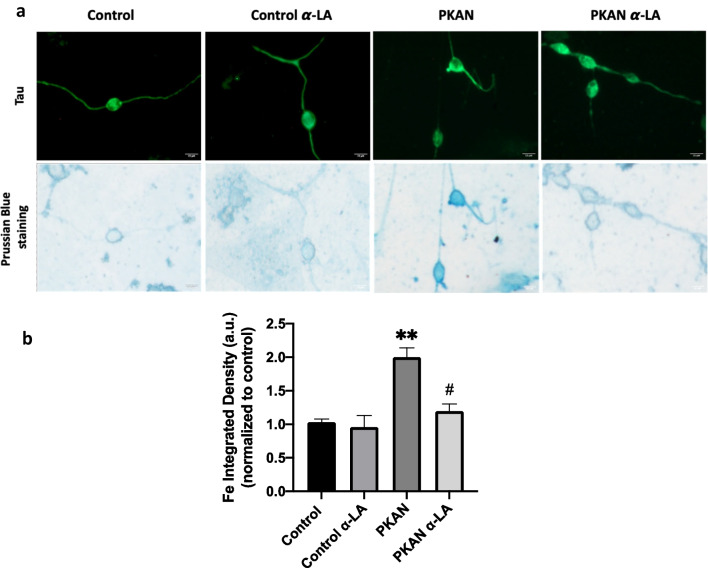
Effect of α-LA on iron accumulation in PKAN induced neurons (iNs). Control and PKAN iNs (P1) were treated with α-LA at 10 μM for 15 days **a** Representative images of iron accumulation by Prussian Blue staining in α-LA treated and untreated control and PKAN iNs. Scale bar = 15 μm. **b** Quantification of iron levels by FIJI-ImageJ. iNs showed positive immunoreactivity against Tau. Data represent the mean ± SD two separate experiment (50 cell images for each condition). **p < 0.01between PKAN patients and controls. ^#^*p* < 0.05 between untreated and treated fibroblasts. A.U., arbitrary units

Next, the beneficial effect of α-lipoic acid in mutant PANK2 induced neurons derived from P1 fibroblasts, which respond positively to α-LA supplementation, was evaluated by examining iron accumulation using Prussian Blue staining. PANK2 mutant induced neurons showed increased Prussian Blue staining indicating iron accumulation. As expected, iron accumulation was eliminated after 10 μM α-LA treatment (Fig. 13a, b).

**Fig. 14:**
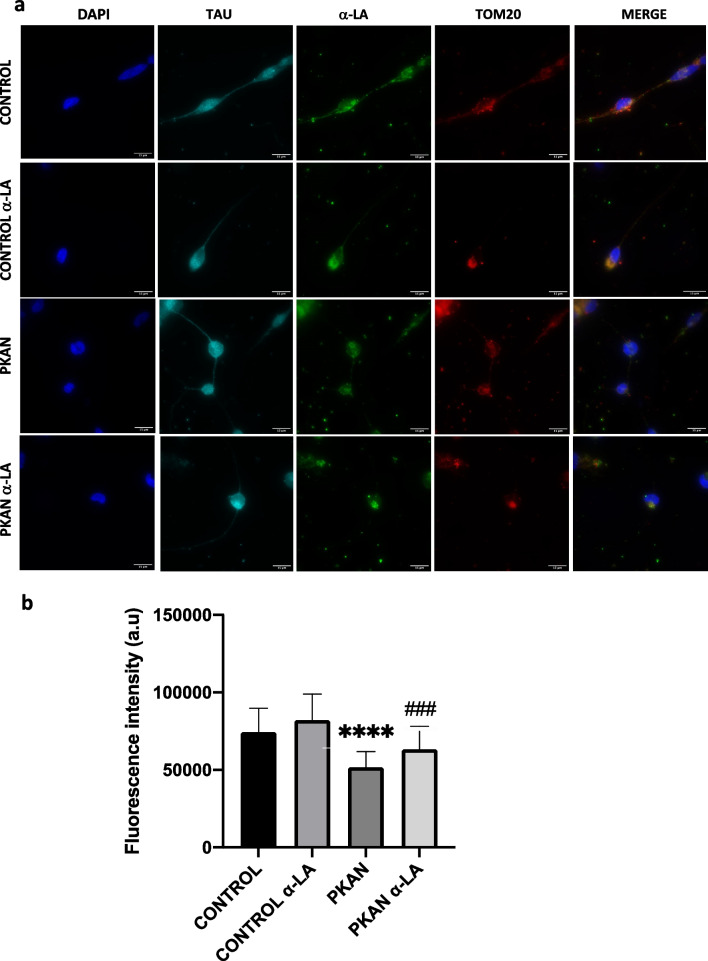
Effect of α-LA on protein lipoylation levels on induced neurons (iNs). Control and PKAN iNs (P1) were treated with α-LA at 10 μM for 15 days **a** Representative images of protein lipoylation levels in α-LA treated and untreated control and P1 PKAN iNs. Scale bar = 15 μm. **b** Quantification of fluorescence intensity by FIJI-ImageJ. iNs showed positive immunoreactivity against Tau. Data represent the mean ± SD of 50 cell images for each condition. ***p < 0.0001between PKAN patients and controls. ^###^*p* < 0.001 between untreated and treated fibroblasts. A.U., arbitrary units

To corroborate the beneficial effect of α-LA treatment in iNs, protein lipoylation was addressed. As shown in Fig. 14a and b, α-LA treatment was able to significantly increase the expression of lipoylated proteins in mutant PANK2 induced neurons.

## Discussion

In this study, we evaluated the effect of α-LA treatment on the pathological alterations in two cellular models of PKAN disease: Skin fibroblasts derived from three PKAN patients and iNs obtained by direct reprogramming.α-LA is a pleiotropic compound required for cell growth, mitochondrial activity and coordination of fuel metabolism as well as regulating gene transcription [57–59]. It is synthesized de novo using intermediates from mtFAS II, S-adenosylmethionine and iron-sulfur cluster [59]. α-LA is characterized by an antioxidant power; it is the reason why it has neuroprotective and anti-inflammatories properties [60]. In this sense, α-LA can reduce pro-inflammatory factors and scavenge ROS and reactive nitrogen species (RNS), as well as restore mitochondrial function and reduce cellular damage [61]. Furthermore, it has been shown that α-LA reduces lipid peroxidation and increases cellular antioxidant activity [62]. α-LA also acts as a cofactor of pyruvate dehydrogenase (PDH), α-ketoglutarate dehydrogenase (KDH), branched-chain ketoacid dehydrogenase and H-protein of the glycine cleavage system (GCS) [27, 63, 64]. In addition, several studies have shown that α-LA also has a chelating effect on metals like iron or copper reducing iron accumulation in the cerebral cortex and a positive impact on oxidative stress [65].

For all the above-mentioned reasons α-LA is a promising candidate for treatment of neurodegenerative diseases such as PKAN.

Given that PKAN is characterized by intracellular iron accumulation, we first assessed α-LA effect on iron homeostasis in PKAN fibroblasts and iNs. The results showed that α-LA supplementation reduced significantly iron accumulation both in fibroblasts and iNs cellular models with residual PANK2 expression levels. These results are consistent with the reduction of age-associated iron accumulation observed in rat cerebral cortex under α-LA treatment [66]. α-LA also prevented iron accumulation induced by ferric ammonium citrate in a zebrafish model [67].

As previously reported by our group, *PANK2* transcripts and protein expression levels are downregulated in PKAN fibroblasts [12]. A reduction of PANK2 protein levels has been also observed in other studies using fibroblasts and neurons [14, 68]. Our findings revealed that α-LA supplementation increases several transcriptions factors -FOXN4, hnRNPA/B and NYA- that activate *PANK2* transcription [44] and restores PANK2 protein expression levels in responsive cell lines.

The partial correction of PANK2 expression levels is supposed to increase CoA biosynthesis allowing the 4′- phosphopantetheinylation of essential mitochondrial proteins such as mtACP [50]. Our results confirmed that several 4′-phosphopantetheine-carrier protein expression levels were indeed increased in responsive pathogenic variants after α-LA supplementation.

It has been demonstrated that mtACP is involved in several crucial processes such as mtFAS II, Fe-S cluster assembly or mitochondrial respiratory complexes assembly [69]. In addition, other studies have reported a decreased oxygen consumption rate in PKAN patient-derived fibroblasts [43]. Likewise, decreased complex I activity has been described in PKAN cellular models [12]. In this work, we observed a positive effect of α-LA supplementation on respiratory complex I activity and increased expression levels of complex I subunits in PKAN fibroblasts. The positive bioenergetics effect of α-LA on PKAN cells is consistent with previous studies in which α-LA was also able to reduce the effect of nitric oxide (NO) excess on oxygen consumption rate (OCR) and ATP production in primary aortic endothelial cells from C57BL/6 J mice [70].

On the other hand, as mtACP also participates in iron-sulfur cluster assembly [50], we evaluated the effect of α-LA supplementation on the downregulated proteins involved in iron-sulfur cluster assembly and aconitase activity, an iron-sulfur dependent enzyme. Our results showed that iron-sulfur cluster metabolism and iron-sulfur group-dependent enzyme activities were restored in PKAN responsive fibroblasts after α-LA supplementation.

Disruption in type II mtFAS and iron-sulfur biogenesis affect α-LA production and consequently protein lipoylation in mutant PKAN cells [12, 59]. α-LA treatment on PKAN cellular models restored PDH and KGDH lipoylation as well as PDH activity. These findings suggest that α-LA can activate PDH and KGDH through mtFAS reestablishment. However, studies have described that α-LA also inhibits pyruvate dehydrogenase kinase (PDK) and hence, increases PDH activity [71–73]. Even though further investigation is needed to understand how α-LA inhibits PDK. In addition, it has been reported that α-LA protects these lipoylated enzymes from inactivation by ROS and 4-hydroxy-2-nonenal (HNE), the main product of lipid peroxidation [74–76].

Lipid peroxidation, lipofuscin accumulation or protein oxidation are consequences of oxidative stress. Our results indicated that α-LA attenuates these physiopathological alterations in responsive PKAN fibroblasts with residual PANK2 expression levels. Clinical trials support that α-LA administration reduces malondialdehyde (MDA) in serum, a biomarker of lipid peroxidation [65] and it can also decrease the alcohol-induced lipid peroxidation and protein oxidation [77].

Regarding the α-LA effect on protein oxidation, there are different parameters to access the protein oxidation level such as protein hydroperoxides (POOH), protein carbonyl groups [78] and the content of protein thiol groups (P-SH). α-LA has a positive effect on human serum albumin oxidation decreasing POOH, and PCO and increasing P-SH. However, a high concentration of α-LA could have a protein prooxidant effect in human serum albumin [79].

Besides being a scavenger of ROS deactivating various free radicals such as the superoxide anion (O_2_^−^), the hydroxyl radical (^•^OH), singlet oxygen (^1^O_2_), peroxynitrite (ONOO^–^), and hypochlorous acid (HClO) [80], our work also showed that α-LA was able to regenerate antioxidant proteins such as SOD or GPX in PKAN fibroblasts. Corroborating this, several studies observed an increase in SOD, GPx and catalase expression after α-LA supplementation in diabetic rats as well as in hemodialysis patients [81, 82]. In turn, GSH also protects cells from oxidative stress, reducing ROS and inhibiting lipid peroxidation. Interestingly, it has been observed that GSH/GSSG ratio is reduced in neurodegenerative diseases and it can be restored by α-LA treatment [83, 84].

The positive effect of α-LA was also confirmed on iNs obtained by direct reprogramming. Our results showed that α-LA supplementation reduces iron accumulation and increases protein lipoylation levels in iNs. Other studies have demonstrated a neuroprotective effect of α-LA in dopaminergic neurons of Parkinson’s disease model [85] and in brain tissues of diabetic rats [86].

It is well known that α-LA is one of the most efficient antioxidants [61] due to its good bioavailability, blood–brain barrier crossing ability and lack of toxic effects at therapeutic doses. Many clinical studies proved beneficial effect of α-LA in many pathological conditions such as diabetes, atherosclerosis, heart diseases, cataract, and neurodegenerative diseases [87].

In addition, as mentioned before, α-LA acts as antioxidant to directly scavenge almost all forms of free radicals (oxygen and nitrogen), chelate transition and heavy metal ions and mediate the recycling of other endogenous antioxidants such as vitamin E, glutathione, and ascorbate [26]. Furthermore, α-LA modulates various signalling cascades either by receptor mediated or non-receptor-mediated processes [61]. However, further studies are needed to clarify if the positive effect of lipoic acid on PKAN with residual expression levels is due to the activation of specific pathways.

However, taking into account that α-LA supplementation has no effect on iron accumulation or lipid peroxidation in PKAN fibroblasts with truncated PANK2 expression levels, it is unlikely that the positive effect of this organosulfur compound was due to its direct chelating or antioxidant properties. In contrast, our results suggest that α-LA supplementation, through the activation of *PANK2* transcription, induces the partial restoration of PANK2 enzyme and mtACP protein levels. For this reason, α-LA treatment is only beneficial in PANK2 pathogenic variants with residual PANK2 expression levels.

## Conclusion

α-LA exhibits significant antioxidant activity in several diseases, including neurodegenerative disorders. In our work, we show that α-LA supplementation can improve the pathological alterations in cellular models of PANK2 pathogenic variants with residual PANK2 expression by a mechanism involving the up-regulation of *PANK2* transcription and enzyme expression levels.

If the positive effect of α-LA is due to a direct antioxidant effect or indirectly stimulates critical transcriptions factor regulating *PANK2* expression are key questions which need more investigation. Likewise, further studies and controlled clinical trials are required to assess the clinical benefit of α-LA in PKAN.

## Supplementary Information

**Additional file 1**. Supplementary figures.

## Data Availability

Data and material are available under request.

## Abbreviations

AASDHPPT: L-aminoadipate-semialdehyde dehydrogenase-phosphopantetheinyl transferase
AASS: Alpha-aminoadipic semialdehyde synthase
ACP: Acyl carrier protein
α-LA: α-Lipoic acid
ALDH1L2: Mitochondrial 10-Formyltetrahydrofolate Dehydrogenase
CoA: Coenzyme A
DAPI: 4',6-Diamidino-2-fenilindol
10-FTHFDH: 10-Formyltetrahydrofolate dehydrogenase
FASII: Fatty acid synthesis type II
FOXN4: Forkhead box protein N4
GPX: Glutathione peroxidase
GSH: Glutathione synthetase
hnRNPA/B: Nuclear ribonucleoprotein A/B
KGDH: Alpha-ketoglutarate
LYRM4: LYR motif containing 4
MTND1: NADH-ubiquinone oxidoreductase chain 1
NBIA: Neurodegeneration with brain iron accumulation
NDUFA9: NADH dehydrogenase (ubiquinone) 1 alpha subcomplex subunit 9
NFS1: NFS1 cysteine desulfurase
NRF2: Nuclear factor erythroid 2-related factor 2
NF-Y: Nuclear transcription factor Y
PANK2: Pantothenate kinase 2
PDH: Pyruvate dehydrogenase
PDK: Pyruvate dehydrogenase kinase
PLAN: PLA2G6-associated neurodegeneration
PLA2G6: Phospholipase A2 group VI
PKAN: Pantothenate kinase-associated neurodegeneration
REST: RE1-silencing transcription factor
RNS: Reactive nitrogen species
ROS: Reactive oxygen species
SOD: Superoxide dismutase
