# Supplementary material for: Alpha-lipoic acid supplementation corrects pathological alterations in cellular models of pantothenate kinase-associated neurodegeneration with residual PANK2 expression levels
Source: Orphanet J Rare Dis. 2023 Apr 12;18:80. doi: 10.1186/s13023-023-02687-5 (PMC10091671; doi:10.1186/s13023-023-02687-5)
Supplement: Supplementary file 1 — Additional file 1. Supplementary figures. [file 13023_2023_2687_MOESM1_ESM.pdf]

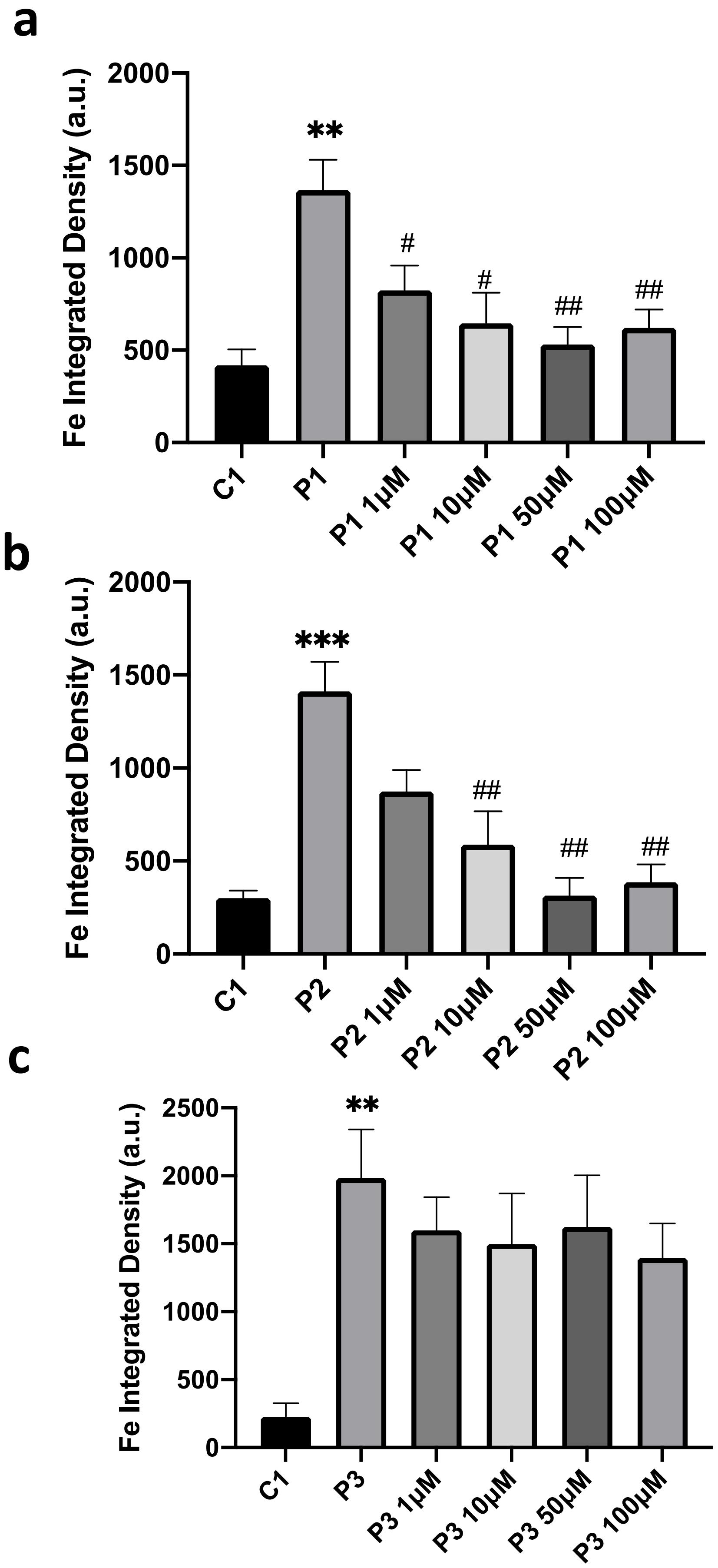

Supplementary figure 1. Quantification of the effect of α-LA on iron accumulation in three PKAN fibroblast cell lines (a) Iron accumulation quantification in control and P1 fibroblasts treated with increasing doses of lipoic acid. **(b)** Quantification of iron levels in control and P2 fibroblasts treated with increasing doses of lipoic acid **(c)** Iron levels quantification of control and P3 fibroblasts treated with increasing doses of α-LA. Data represent the mean±SD of three separate experiments. **$p<0.01$, ***$p<0.005$ between PKAN patients and controls. $^{\#}p<0.05$, $^{\#\#}p<0.01$ between untreated and treated fibroblasts. A.U., arbitrary units.
